# Enhancing the Stability and Initial Coulombic Efficiency of Silicon Anodes through Coating with Glassy ZIF-4

**DOI:** 10.3390/nano14010018

**Published:** 2023-12-20

**Authors:** In-Hwan Lee, Yongsheng Jin, Hyeon-Sik Jang, Dongmok Whang

**Affiliations:** 1 Department of Advanced Materials Science and Engineering, Sungkyunkwan University (SKKU), Suwon 16419, Republic of Korea; koggiree24@skku.edu (I.-H.L.); jinzuyeye@skku.edu (Y.J.); 2 School of Semiconductor Science & Technology, Jeonbuk National University, Jeonju 54896, Republic of Korea

**Keywords:** glassy MOF, glass transformation, silicon anode, initial coulombic efficiency

## Abstract

The high capacity of electrodes allows for a lower mass of electrodes, which is essential for increasing the energy density of the batteries. According to this, silicon is a promising anode candidate for Li-ion batteries due to its high theoretical capacity. However, its practical application is hampered by the significant volume expansion of silicon during battery operation, resulting in pulverization and contact loss. In this study, we developed a stable Li-ion anode that not only solves the problem of the short lifetime of silicon but also preserves the initial efficiency by using silicon nanoparticles covered with glassy ZIF-4 (SZ-4). SZ-4 suppresses silicon pulverization, contact loss, etc. because the glassy ZIF-4 wrapped around the silicon nanoparticles prevents additional SEI formation outside the silicon surface due to the electrically insulating characteristics of glassy ZIF-4. The SZ-4 realized by a simple heat treatment method showed 74% capacity retention after 100 cycles and a high initial efficiency of 78.7%.

## 1. Introduction

The increasing demand for electric vehicles and sustainable energy storage systems has heightened the need for advanced lithium-ion batteries (LIBs) with superior energy capacity and longevity [1]. Graphite is currently the dominant anode material in LIBs, but its limited theoretical capacity (372 mAh g^−1^) restricts the potential energy density enhancement of the battery [1]. In contrast, silicon (Si), with a high theoretical capacity (4200 mAh g^−1^), a favorable Li-uptake potential, and natural abundance, emerges as a potential successor for next-generation anodes [2,3,4]. However, significant challenges need to be addressed for the widespread application of silicon materials as battery anodes. The most pressing issue is its large volume expansion (up to 300–400%) during the lithiation process [5], which can lead to mechanical stresses within the anode. This substantial expansion and contraction during charge and discharge cycles can cause the silicon particles to fracture, leading to rapid capacity decay. Moreover, these volume changes can destabilize the solid electrolyte inter-phase, a crucial component for battery stability, resulting in increased internal resistance and further capacity loss [6,7]. Research and development efforts are ongoing to overcome these challenges, and several strategies have been proposed and tested. These include the design of silicon–carbon composites [8], the use of silicon nanostructures [9,10,11,12], the development of flexible and robust binders [13,14,15], and the engineering of electrolytes compatible with silicon anodes [16]. In particular, since silicon with a diameter of less than 150 nm does not easily crack or break during lithiation [6], various silicon nanostructures, such as nanoparticles [9], nanowires [10], nanotubes [11], and nanorods [12], have been extensively studied. However, fabrication of the nanostructures is not cost-effective, and nanostructured silicon alone cannot effectively suppress specific volume changes. An alternative approach to improve the cycling stability of silicon anodes is by forming composites with materials that can accommodate or suppress volume expansion, such as inorganic [17,18,19], organic [20,21], and porous carbon shells [22,23,24]. The inorganic shells, such as metal oxides and nitrides, can alleviate the problem caused by silicon expansion. However, a sacrifice in energy density is inevitable due to the large mass of the inorganic shell. The organic shell has been employed to build a flexible surface coating that can not only tolerate the volume change but also block the penetration of electrolytes into the silicon surface. However, it is difficult to obtain uniform polymer coatings due to disorderly stacked polymer clews and agglomerates, resulting in penetration of the electrolyte toward the silicon surface. The case of porous carbon shells has received a lot of attention due to their electrical conductivity, cost-effectiveness, and flexibility [25]. However, porous carbon has a large surface area that allows for irreversible reactions that cause lithium consumption, dramatically reducing the initial efficiency of the batteries [26,27]. The low initial coulombic efficiency of the batteries reduces the total amount of lithium available throughout the battery, which is critical to the energy density of the battery [28].

Metal–organic frameworks (MOFs) are crystalline materials with high porosity and large free volume and surface area [29]. With these properties, MOFs have been used in various applications, including gas separation, energy storage, and catalysis [30,31,32]. In particular, MOFs have been used in energy storage systems as silicon/MOF-based carbon composites because they mitigate the significant volume expansion of silicon while providing a channel for Li ions to move through [33,34]. However, directly synthesizing MOFs on silicon has drawbacks, like the formation of many defects in the MOFs and a complicated process. Furthermore, the problem of low initial coulombic efficiency due to irreversible lithium consumption still exists due to use of pyrolyzed MOFs.

In this work, we fabricated silicon nanoparticles covered with glassy MOF to solve the electrode degradation problem due to Si volume expansion without additional loss of initial coulombic efficiency. Zeolitic imidazolate framework-4 (ZIF-4), an electrically insulating MOF with zeolite-like topology, undergoes glass transformation around 300 °C, and glassy ZIF-4 can be synthesized by the melt–quench method [35]. Notably, the glassy ZIF-4 shows an excellent Li-ion conductivity of about 1.61 × 10^−4^ S cm^−1^ in the presence of a small amount of solvent, making it suitable as a shell for silicon anodes [36]. In addition, the glassy ZIF-4 can form a consistent shell, and its electrically insulating nature can inhibit additional solid–electrolyte interface (SEI) formation from the shell material. The composite anode, where silicon nanoparticles are covered with glassy ZIF-4, showed a capacity retention of about 74% after 100 cycles due to the glassy ZIF-4 shell and an initial efficiency of 78.7%, about 10% higher than the silicon nanoparticles covered with carbon matrix due to the insulating properties of the glassy ZIF-4 shell.

## 2. Materials and Methods

### 2.1. Materials Synthesis

ZIF-4 was synthesized following the method described in Ref. [37]. Zinc (II) acetate dihydrate (4.4 g, >98%, Sigma-Aldrich, St. Louis, MO, USA) was dissolved in 60 mL of propylamine (98%, DAEJUNG, Sihung, Republic of Korea). Imidazole (2.72 g, >99%, Sigma-Aldrich) was separately dissolved in 120 mL of dimethyl formamidine (DMF, 99.9%, DAEJUNG). Zinc (II) acetate solution was then added dropwise to the imidazole solution while stirring continuously at room temperature. After 4 h of stirring, the resulting mixture was centrifugated using DMF as a solvent 3 times and dried at 80 °C for 24 h.

(i) SZ, (ii) SZ-4, and (iii) SZ-10 indicate (i) the mixture of silicon nanoparticles (50 nm, >99%, Sigma-Aldrich), super-P, and ZIF-4 powder with the ratio mentioned below; (ii) heat-treated SZ in Ar atmosphere at 400 °C for 1 h; and (iii) heat-treated SZ-4 in Ar atmosphere at 1000 °C for 3 h, respectively. SZ was obtained with a mixture ratio of Si nanoparticles (60 wt%), a conducting agent (Super-P, 10 wt%, Alfa Aesar, Tewksbury, MA, USA), and ZIF-4 powder (30 wt%).

### 2.2. Material Characterization

Morphological and elemental analysis of the samples was conducted using a field emission scanning electron microscope (FESEM, JEOL JSM-7600F, Tokyo, Japan, 15 kV) equipped with an energy-dispersive X-ray spectroscopy (EDS) detector at the MEMS·Sensor Platform Center of Sungkyunkwan University (SKKU). The crystalline phase of the materials was determined by X-ray diffraction (XRD, D8 ADVANCE, Bruker, Waltham, MA, USA) with Cu-Kα (λ = 1.540 Å) radiation. Thermogravimetric analysis (TGA) was conducted on TG/DTA 7300 from room temperature to 1000 °C in an N_2_ environment. Differential scanning calorimetry (DSC) was performed on Nexta DSC 600 from room temperature to 500 °C in N_2_.

### 2.3. Electrochemical Characterization

Electrochemical studies of SZ, SZ-4, and SZ-10 were carried out using coin-type cells (CR2032, stainless steel, Welcos, Seoul, Republic of Korea) assembled with Li metal (Alfa Aesar) as a counter electrode. The coin-type cells were assembled in an argon-filled glove box (KOREA KIYON, Seoul, Republic of Korea) with a polymer separator (PP/PE/PP, 20 μm, Celgard 2320, Charlotte, NC, USA). Before cell assembly, all electrodes were fabricated using a slurry composed of a mixture of powder (75 wt%), a conducting agent (Super-P, 10 wt%), and a binder (sodium alginate, SA, 15 wt%, Alfa Aesar) in N-methyl-2-pyrrolidine (NMP, 99.7%, DAEJUNG). This slurry was then coated onto thin copper foil (9 μm, MTI KOREA, Yeosu-si, Republic of Korea) using a conventional casting. The loading amount of the electrode was controlled to be 1.0 mg cm^−2^. The coated foils were dried in a vacuum oven at 80 °C overnight. The electrolyte was a 1 M solution of LiPF_6_ (>99.99%, Sigma-Aldrich) in a 1:1 volume ratio of ethylene carbonate (EC, 98%, Sigma-Aldrich) and diethyl carbonate (DEC, >99%, Sigma-Aldrich), with an addition of 5% fluoroethylene carbonate (FEC, 99%, Sigma-Aldrich). All electrochemical tests were performed using potentiostat (WonATech-WBCS 3000, Ltd., Seoul, Republic of Korea). Electrochemical impedance spectroscopy (EIS) for the cells was performed over a 10^6^ to 0.01 Hz frequency range. Fitting of EIS data was conducted by using Nova, Harlingen, TX, USA.

## 3. Results and Discussion

**Figure 1:**
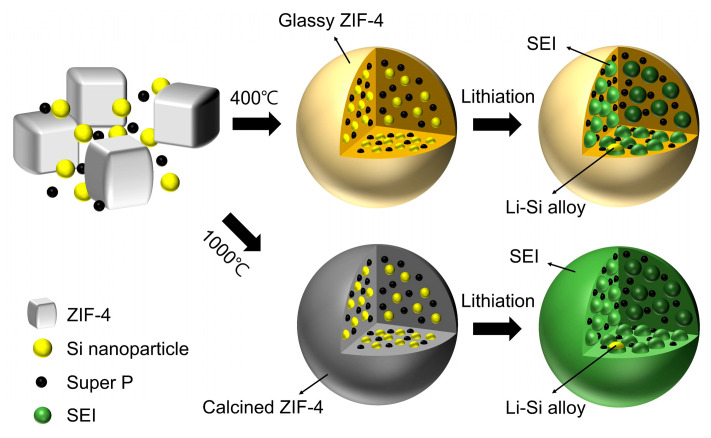
Schematic illustration of fabrication and lithiation process for silicon nanoparticles covered with glassy ZIF-4 (SZ-4) and silicon nanoparticles coated with carbon matrix (SZ-10).

Crystalline ZIF-4 undergoes a phase change to a glassy state upon heating above its glass transition temperature (296 °C) in an inert gas environment [35]. The Li-ion conductivity of this glassy ZIF-4 is sufficiently high, allowing its use as a quasi-solid-state electrolyte in the presence of some liquid electrolytes [36]. To effectively stabilize the silicon anode, a shell that can prevent degradation due to volume expansion of the silicon is required, and the shell should not have irreversible lithium consumption to form SEI. Glassy ZIF-4 would be a promising candidate for the shell because of its impressive electrochemical stability and high Li-ion conductivity [36,38]. The SZ-4 was realized by mixing ZIF-4 with silicon nanoparticles and super P and heating it above the glass transition temperature of ZIF-4. As with the studies that coated silicon with an electrically insulating shell [39,40], this structure can regulate the pulverization of silicon and contact loss and increases the initial efficiency without additional lithium consumption due to the insulating characteristics of the glass ZIF-4 shell (Figure 1). Meanwhile, SZ-10 also can deal with the volume expansion and contact loss of the silicon anode. Still, a significant initial efficiency loss cannot be avoided due to irreversible lithium consumption on the surface of the carbon shell, which has a large surface area.

**Figure 2:**
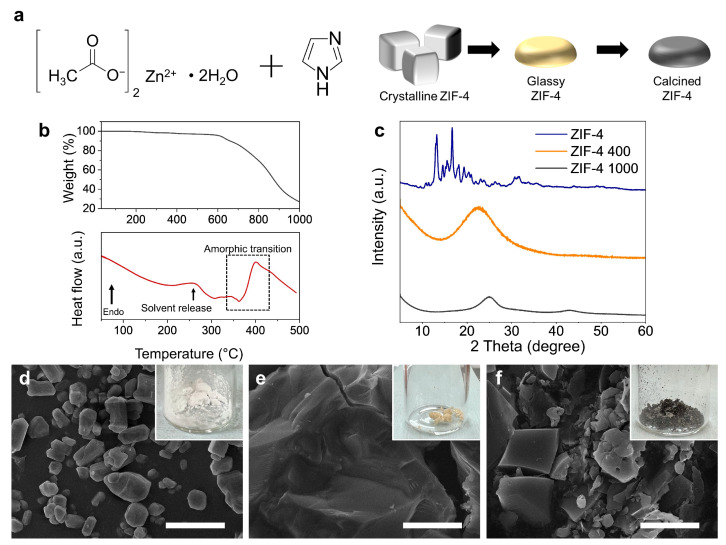
(**a**) Schematic illustration of ZIF-4 precursors and ZIF-4′s phase transition process. (**b**) TGA (upper graph) and DSC (lower graph) of ZIF-4 with increasing temperature at N_2_ atmosphere. (**c**) XRD spectra of ZIF-4 with three different heat treatment conditions. (**d**–**f**) SEM image of ZIF-4, ZIF-4 400, and ZIF-4 1000. The insets of (**d**–**f**) indicate optical images of each material. Scale bar, 10 μm.

ZIF-4 was obtained as white crystalline powders by reacting with zinc acetate and imidazole precursors (Figure 2a,d) [37,41]. First, the glass transition and calcination temperatures of ZIF-4 were investigated using TGA and DSC (Figure 2b). The TGA spectrum shows that ZIF-4 began to decompose at 600 °C in an N_2_ atmosphere, leaving only about 27% of the mass at 1000 °C (upper graph of Figure 2b). From 600 °C, calcination occurs, which turns ZIF-4 into carbon matrix. To confirm the occurrence of vitrification, differential scanning calorimetry (DSC) was employed to monitor the enthalpic responses to the phase transition processes. ZIF-4 crystals undergo three enthalpy responses: (i) the endotherm caused by solvent release (256 °C), (ii) an exotherm due to the formation of a low-density amorphous phase (362 °C), and (iii) an endotherm due to transformation from the low-density amorphous phase into a high-density amorphous phase (400 °C). According to the DSC curve, the ZIF-4 that was annealed at 400 °C became an amorphous ZIF-4 because this is the temperature at which the amorphic transition occurs (lower graph of Figure 2b). To verify that a phase change of ZIF-4 had indeed occurred, XRD analysis was performed (Figure 2c). In the XRD spectrum, the as-synthesized ZIF-4 without heat treatment shows the same sharp peaks at 11, 13.5, 14.8, 15.5, and 17.7° as in the previous study [42]. On the other hand, after heating at 400 °C, the sharp diffraction peaks of ZIF-4 disappeared. This indicates that ZIF-4 has undergone a phase change above the glass transformation temperature, resulting in glassy ZIF-4. The XRD spectrum of ZIF-4 which is heat-treated at 1000 °C exhibited two broad and low-intensive diffraction peaks centered at 2θ = 20°–26° and 43°–44°, which can be attributed to the (002) and (100) diffractions of the hexagonal graphitic carbon lattice, clearly indicating a lack of long-range-order nature and a low graphitization degree of the carbon materials [43,44]. The morphology change of ZIF-4 with the heat treatment temperature was also confirmed by SEM (Figure 2d–f). As-synthesized ZIF-4 are 1–10 μm sized crystalline powders with polyprism morphology (Figure 2d). On the other hand, the glassy ZIF-4 heat-treated at 400 °C shows a morphology change and merging of the powders, resulting in solids with a size of more than tens of micrometers (Figure 2e). This indicates that ZIF-4 has liquid-like behavior during glass transformation. The carbon matrix formed by heat-treating glassy ZIF-4 at 1000 °C exhibits a crumbly character and exists as a powder with a size of about 10 μm (Figure 2f). The color of the powder changed to white (0 °C, as-synthesized ZIF-4), yellow (400 °C, glassy ZIF-4), and black (1000 °C, carbon matrix) depending on the heat treatment temperature (inset of Figure 2d–f).

**Figure 3:**
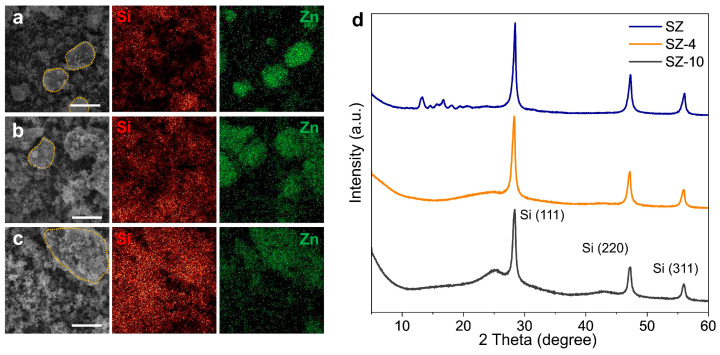
SEM image and EDS mapping image of (**a**) SZ, (**b**) SZ-4, and (**c**) SZ-10. (**d**) XRD spectra of SZ (blue line), SZ-4 (orange line), and SZ-10 (gray line). Scale bar, 10 μm.

To fabricate the silicon composite materials, silicon nanoparticles were mixed with ZIF-4 and super-P, and the elemental distribution and crystallinity changes were analyzed after heat treatment (Figure 3). For the SZ sample, which is a mixture of silicon, ZIF-4, and super-P, EDS mapping shows no elemental distribution matching for Si and Zn (Figure 3a). It should be clear to understand with the part inside the dashed orange line. Since Zn represents ZIF-4, the mismatch of Si and Zn indicates that ZIF-4 and silicon nanoparticles are separated. On the other hand, for SZ-4, where SZ was heat-treated at 400 °C, Zn is observed where Si is present, suggesting that the glassy ZIF-4 covered the silicon nanoparticles because of the good wettability of melted ZIF on the silicon oxide surface [45] (Figure 3b). Similar to SZ-4, the EDS mapping of SZ-10, where SZ-4 was heat-treated at 1000 °C, shows a matching distribution of Zn and Si (Figure 3c). This indicates that the carbon matrix and silicon nanoparticles did not separate during calcination. XRD analysis confirmed that the same glass transformation and calcination occurred in the mechanical mixture of silicon nanoparticles and super-P as in pure ZIF-4 (Figure 3d). Similar to pure ZIF-4, SZ also lost the diffraction peaks in the XRD spectrum after 400 °C heat treatment, and a carbon peak which has a lack of long-range order was observed after 1000 °C heat treatment. In addition, peaks corresponding to the (111), (220), and (311) faces of silicon are observed, indicating that there is no change in the crystalline structure of silicon nanoparticles during the heat treatment process.

**Figure 4:**
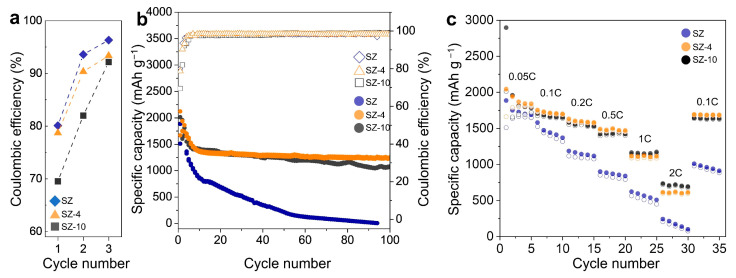
(**a**) First 3 cycles’ Coulombic efficiency of SZ, SZ-4, and SZ-10 electrodes at 0.05 C. (**b**) Cycling stability and Coulombic efficiency of the three electrodes at 0.05 C for first the 3 cycles and at 0.5 C for the rest. (**c**) Rate capability of three electrodes at 0.05, 0.1, 0.2, 0.5, 1, 2, and 0.1 C.

To check the electrochemical performance of SZ, SZ-4, and SZ-10, a coin-type cell with Li metal as a counter electrode was fabricated and evaluated (Figure 4). The first three cycles were performed at 0.05 C to stabilize the electrodes, and the Coulombic efficiency of each electrode was compared (Figure 4a). In the case of SZ, the formation of SEI on the surface of silicon nanoparticles was induced, resulting in an initial efficiency of 80% and a Coulombic efficiency of 93.6 and 97.1% in the 2nd and 3rd cycles, respectively. In the case of SZ-4, the initial efficiency was 78.7%, similar to SZ, and the Coulombic efficiency was 90.3 and 93.3% in the 2nd and 3rd cycles, respectively. This indicates that no additional irreversible lithium consumption occurs in the glassy-ZIF-4-covered structure. On the other hand, SZ-10 has a carbon matrix with a large surface area and electrical conductivity, so irreversible lithium consumption occurred on the carbon matrix surface, resulting in an initial efficiency of 69.5%, much lower than for SZ and SZ-4, and Coulombic efficiency of 82.0 and 92.2% in the 2nd and 3rd cycles, respectively. The Coulombic efficiencies in the 3rd cycle were similar for all three electrodes, indicating that the SZ-10 sample did not have low initial efficiency due to structural defects but rather due to the SEI formed by the irreversible lithium consumption on the carbon matrix surface at the 1st cycle.

After the first three cycles at 0.05 C, the cycling stabilities of three electrodes were evaluated at 0.5 C (Figure 4b). The SZ electrode showed an initial discharge capacity of 1510 mAh g^−1^, followed by rapid capacity fading to a discharge capacity of only 217 mAh g^−1^ at the 50th cycle. The SZ-10 electrode showed a much larger first charge capacity of 2897 mAh g^−1^, but, as a result of the irreversible reaction by the carbon shell, the first discharge capacity was significantly reduced to 2013 mAh g^−1^. The SZ-10 electrode showed higher cycling stability than the SZ electrode due to the structural stability of the carbon shell, showing a discharge capacity of 1068 mAh g^−1^ at the 100th cycle, indicating a capacity retention of 53.1%. On the other hand, the SZ-4 electrode showed an initial charge capacity of 2122 mAh g^−1^. It also showed stable cycling stability without significant irreversible reactions in the early cycles, resulting in an initial discharge capacity of 1669 mAh g^−1^. It also showed a discharge capacity of 1250 mAh g^−1^ at the 100th cycle, showing a capacity retention of 74%. To evaluate the rate capability of the electrodes, new batches of SZ, SZ-4, and SZ-10 electrodes were performed for five cycles at 0.05, 0.1, 0.2, 0.5, 1, 2, and 0.1 C (Figure 4c). For the SZ electrode, the structure of the electrode should be the least affected by the C rate, but the low cycling stability of the electrode made an accurate assessment difficult. The SZ-4 electrode showed no significant difference in capacity reduction at high current densities when compared to the SZ-10 electrode. This indicates that the glassy ZIF-4 does not significantly hinder Li-ion transport.

**Figure 5:**
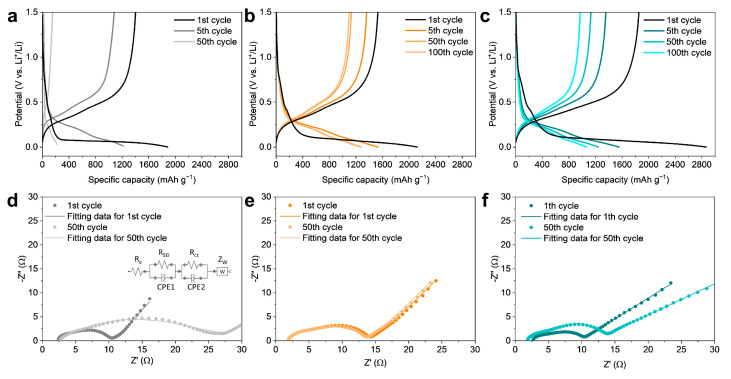
Voltage profile of the (**a**) SZ, (**b**) SZ-4, and (**c**) SZ-10 electrodes at various cycles. The Nyquist plots of the (**d**) SZ, (**e**) SZ-4, and (**f**) SZ-10 electrodes after the 1st cycle and 50th cycle.

The voltage profile of each electrode was used to accurately evaluate the initial efficiency and cycling stability (Figure 5a–c). As seen earlier, SZ-10 showed significantly lower discharge capacity than its charge capacity, resulting in the lowest initial efficiency among the three electrodes (Figure 5c). At the 1st cycle of each electrode, the discharge curves display a long flat plateau below 0.1 V due to the fact that crystalline silicon becomes an amorphous Li-Si alloy during the lithiation (Figure S1).

c-Si + xLi → a-Li_x_Si, (1)

At the 5th cycle and beyond, however, the electrodes start to react in the theoretical reaction voltage region of the silicon. Additionally, the fifth lithiation dQ/dV curve shows a very weak peak at about 0.08 V and a peak at 0.24 V (Figure S1). The peak at 0.24 V is due to the amorphous silicon becoming an amorphous Li-Si alloy. The stabilities at the interface of three electrodes were studied by electrochemical impedance spectroscopy (EIS) (Figure 5d–f). Figure 5d–f demonstrate the Nyquist plots of the SZ, SZ-4, and SZ-10 electrodes after the 1st and 50th cycles. The equivalent circuit model used for fitting the Nyquist plots is shown in the inset of Figure 5d. In the circuit model, R_e_ represents the contributions from the ionic conduction in the electrolyte and external electrical contact resistances. R_SEI_ and R_ct_ are the resistance of the SEI film and the charge-transfer resistance of the electrode/electrolyte interface, respectively. Z_w_ is the Warburg impedance corresponding to the lithium-diffusion process. For the SZ, SZ-4, and SZ-10 electrodes, R_e_ almost does not change after the 50th cycle (SZ: 2.06 to 2.69 Ω, SZ-4: 1.69 to 1.72 Ω, SZ-10: 2.45 to 1.79 Ω). This indicates that there was no degradation of the current collector or depletion of the electrolyte. In the case of the SZ electrode, the R_SEI_ and R_ct_ of the SZ electrode significantly increase after the 50th cycle (R_SEI_: 2.49 to 9.06 Ω, R_ct_: 4.88 to 15.4 Ω) (Figure 5d). This indicates that the SEI film was thickened during cycling due to the side reaction of electrolytes. In the case of the SZ-4 electrode, the R_SEI_ and R_ct_ after the 50th cycle are similar to those after the 1st cycle (R_SEI_: 4.69 to 4.61 Ω, R_ct_: 5.36 to 5.23 Ω), showing superior cycling stability of the SZ-4 electrode. On the other hand, the R_SEI_ and R_ct_ of the SZ-10 electrode increase after the 50th cycle (R_SEI_: 2.57 to 4.06 Ω, R_ct_: 4.97 to 7.39 Ω). The cycling stability of the SZ-10 electrode is also improved, but not as much as for the SZ-4. The Warburg coefficient and diffusivity (D_Li+_) were calculated from Z_w_ (Table S1). According to the results, the calculated D_Li+_ values of all electrodes are similar (~10^−11^ cm^2^ S^−1^), indicating that the active materials of all electrodes are Si nanoparticles [46,47,48,49].

## 4. Conclusions

A stable Li-ion anode, silicon nanoparticles covered with glassy ZIF-4, was realized by a simple thermal annealing method of silicon nanoparticles and a ZIF-4 and super-P mixture. The glassy ZIF-4 regulates pulverization and contact loss of silicon due to volume expansion during charge and discharge, improving cycling stability. In addition, unlike the carbon matrix typically used to regulate the volume expansion problem of silicon, the glassy ZIF-4 showed high initial Coulombic efficiency without additional irreversible reactions, resulting from its electrically insulating nature. With these characteristics, the SZ-4 electrode showed 74% capacity retention after 100 cycles and a high initial efficiency of 78.7%. In addition, it is further confirmed that the improved cycling stability could be attributed to the coating of glassy ZIF-4 by using EIS analysis. This strategy will advance both the cycling stability and initial Coulombic efficiency of silicon anodes, providing a new approach to designing practical silicon anodes for high-energy-density Li-ion batteries.

## Data Availability

Data underlying the results presented in this paper are not publicly available at this time but may be obtained from the authors upon reasonable request.

## Supplementary Materials

The following supporting information can be downloaded at: https://www.mdpi.com/article/10.3390/nano14010018/s1. Figure S1. dQ/dV curves of SZ, SZ-4, and SZ-10 electrode obtained from the galvanostatic curves. Table S1. Fitting results of all elements of the equivalent circuit model and Li-ion chemical diffusion coefficient calculated by EIS methods.
