# Supplementary material for: Enhancing the Stability and Initial Coulombic Efficiency of Silicon Anodes through Coating with Glassy ZIF-4
Source: Nanomaterials (Basel). 2023 Dec 20;14(1):18. doi: 10.3390/nano14010018 (PMC10780738; doi:10.3390/nano14010018)
Supplement: Supplementary file 1 [file nanomaterials-14-00018-s001.zip › nanomaterials-2706413-supplementary.pdf]

Supporting Information

Enhancing the Stability and Initial Coulombic Efficiency of Silicon Anodes through Coating with Glassy ZIF-4

In-Hwan Lee [1,†], Yongsheng Jin [1,†], Hyeon-Sik Jang [2,*] and Dongmok Whang [1,*]

[1] Department of Advanced Materials Science and Engineering, Sungkyunkwan University (SKKU),
Suwon 16419, Republic of Korea; koggiree24@skku.edu (I.-H.L.); jinzuyeye@skku.edu (Y.J.)

[2] School of Semiconductor Science & Technology, Jeonbuk National University, Jeonju 54896, Republic of Korea

* Correspondence: hsjang88@jbnu.ac.kr (H.-S.J.); dwhang@skku.edu (D.W.)

† These authors contributed equally to this work.

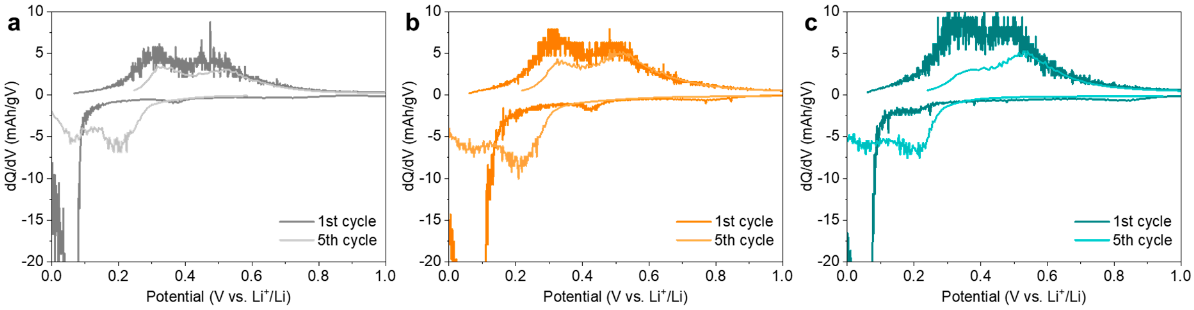

Figure S1. dQ/dV curves of SZ, SZ-4, and SZ-10 electrode obtained from the galvanostatic curves.

Table S1. Warburg coefficient and Li-ion chemical diffusion coefficient calculated by EIS methods.

Electrode	Conditions	Warburg coefficient (Ω $s^{-0.5}$)	Diffusivity (cm^2 s^{-1})
SZ	After 1st cycle	14.684	8.14352E-11
	After 50th cycle	13.755	9.28082E-11
SZ-4	After 1st cycle	23.095	3.29226E-11
	After 50th cycle	21.834	3.6834E-11
SZ-10	After 1st cycle	14.815	8.00045E-11
	After 50th cycle	21.93	3.65124E-11

Diffusivities were calculated from the Warburg element. The formula which is described below was used for calculating diffusivities.

$$D_{Li^+} = \frac{R^2T^2}{2n_{Li^+}^4A^2F^4C_{Li^+}^2\sigma_W^2}$$

In this equation, R represents the gas constant (8.314 J mol^{-1} K^{-1}), T represents temperature in Kelvin (298.15 K), n_{Li+} represents the number of electrons per species reaction (for lithium ions, $n_{Li+} = 1$), A represents the surface area of electrode (1.54 cm^2), F means Faraday constant (96485 C mol^{-1}), C_{Li+} represents the concentration of lithium ions in mol cm^{-3} (0.001 mol cm^{-3}), respectively.
